# Diagnosis of neonatal neurofibromatosis type 1: a case report and review of the literature

**DOI:** 10.1186/s12887-023-04077-z

**Published:** 2023-05-24

**Authors:** Qiuying Zheng, Bei Xia, Xiaoli Zhao, Ruijie Wang, Fusui Xie, Nihui Pei, Hongwei Tao, Tingting Ding, Lei Liu

**Affiliations:** 1 Department of Ultrasound, Shenzhen Children’s Hospital, Shenzhen, China; 2 Neonatal Intensive Care Unit, Shenzhen Children’s Hospital, Shenzhen, China; 3 Radiology Department, Shenzhen Children’s Hospital, Shenzhen, China

**Keywords:** Neurofibromatosis type 1, Neonate, Ultrasound, Imaging, Case report

## Abstract

**Background:**

Neurofibromatosis Type 1 (NF1) is a rare genetic disorder characterized with the development of multiple benign tumors on the nerves and skin.

**Case presentation:**

This report described a neonatal case with a large mass observed on the left side of the maxillofacial and cervical region at birth. Meantime, multiple cafe-au-lait macules (CALMs) were seen on the trunk and both lower extremities.

**Conclusions:**

In this case, the clinical features of the rare NF1 neonate are discussed along with its ultrasound findings.

## Background

As an autosomal dominant disorder, Neurofibromatosis Type 1 (NF1) has a neonatal morbidity of around 1/3000 [1]. NF1 gene mutation is the direct cause of NF1.Located on chromosome 17q11.2, NF1 is a tumor suppressor gene that encodes a neurofibromin, involved in the transduction cascade of multiple signals such as Ras/RAF/MEK/ERK, Akt/mTOR and AC/cAMP, and associated with cell proliferation and differentiation [2]. Typical clinical manifestations of NF1 are cutaneous neurofibromas, cafe-au-lait macules (CALMs), freckles, and Lisch nodules (iris malformations). Some cases are characterized clinically by seizures, learning and cognitive impairments, autism spectrum disorders, vascular diseases, cardiac malformations, central nervous system (CNS) tumors, skeletal abnormalities and other syndromes [3]. Many patients are usually undiagnosed or miss the best treatment opportunity due to the lack of symptoms or late onset. Only 46% of patients without family history are diagnosed before age 2 [4]. Among them, neonatal cases are even rarer. This report discussed a neonatal case with a large mass on the maxillofacial and cervical region, as well as its ultrasound imaging features and clinical presentations.

### Case presentation

**Fig. 1:**
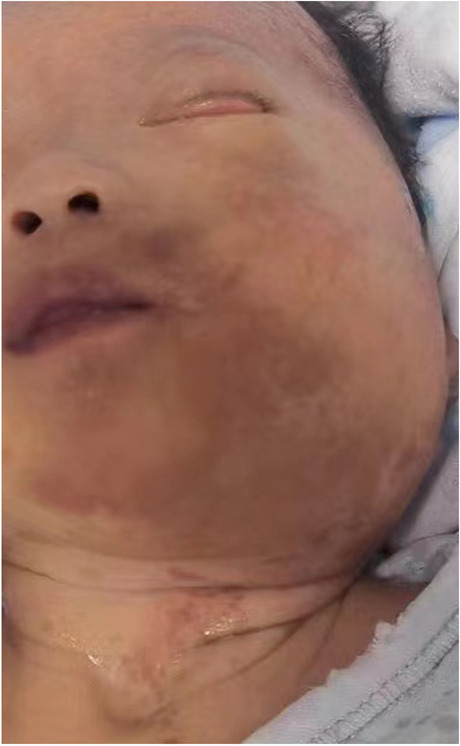
Image of a large mass in the left maxillofacial and cervical region

A 4-day-old female infant with gestational age of 39 weeks was admitted to our hospital with a mass of progressive enlargement in the left maxillofacial region. The child was born naturally due to premature rupture of membranes, with the umbilical cord around the neck one loop, and no abnormality found in placenta and amniotic fluid. Apgar score at birth was 10 in 1 min, 5 and 10 min respectively. Physical examination after admission showed edema on the left facial region and left eyelid region, ectropion of the left eyelid, conjunctival congestion, and faint yellow secretion. The mass on the left side of the maxillofacial to cervical region was slightly tough, with low local skin temperature, average mobility and without rupture (Fig. 1). The number of CALMs seen on the trunk and both lower extremities was more than 6, with the largest greater than 11 cm. The lung respiratory sound was coarse, and sputum sound heard. The family history of the newborn was normal, except that her father suffered from CALMs.

**Fig. 2:**
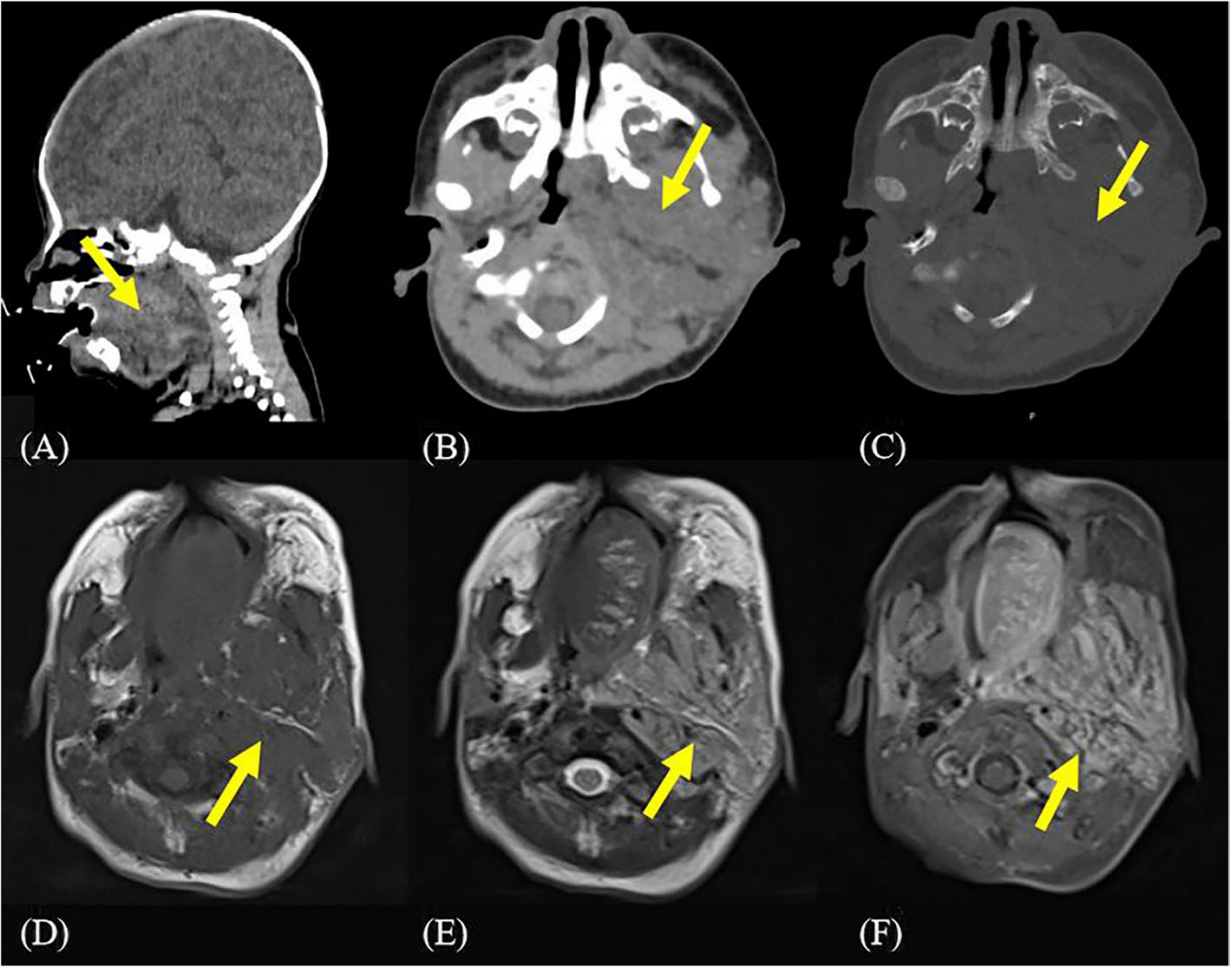
CT & MR images. **A** Sagittal soft tissue window shows a large mass occupying in the left maxillofacial and cervical region (arrow); **B** Coronal soft tissue window shows that the lesion involves the left parapharyngeal space (arrow); **C** Coronal bone window shows localized bone destruction in the left mandible (arrow); **D** Enormous mass occupying in the left maxillofacial and cervical region involves soft tissues such as the parapharyngeal space, with mixed iso-signal in T1-weighted imaging (arrow) and **E** mixed high signals in T2-weighted imaging (arrow); **F** Contrast enhanced MRI shows significantly more uniform enhancement, partly nodular and lumpy enhancement (arrow)

CT showed a large mass occupying in the maxillofacial and cervical region that compressed the airway, involving the left cavernous sinus and locally encircling the optic nerve (Fig. 2A-C). CT suspected neurofibromatosis. MRI also showed an enormous irregular mass in the left maxillofacial and cervical region with unclear borders, encasing the left carotid sheath, oppressing the airway, involving the left parotid gland, the left cavernous sinus, and extending into the left orbit (Fig. 2D, E). Contrast enhanced MRI revealed a more homogeneous enhancement of the mass as a whole, with some nodular and mass-like enhancement (Fig. 2F). For these, the MRI diagnosis was suspected neurogenic tumor or Kaposi’s hemangioendothelioma.

**Fig. 3:**
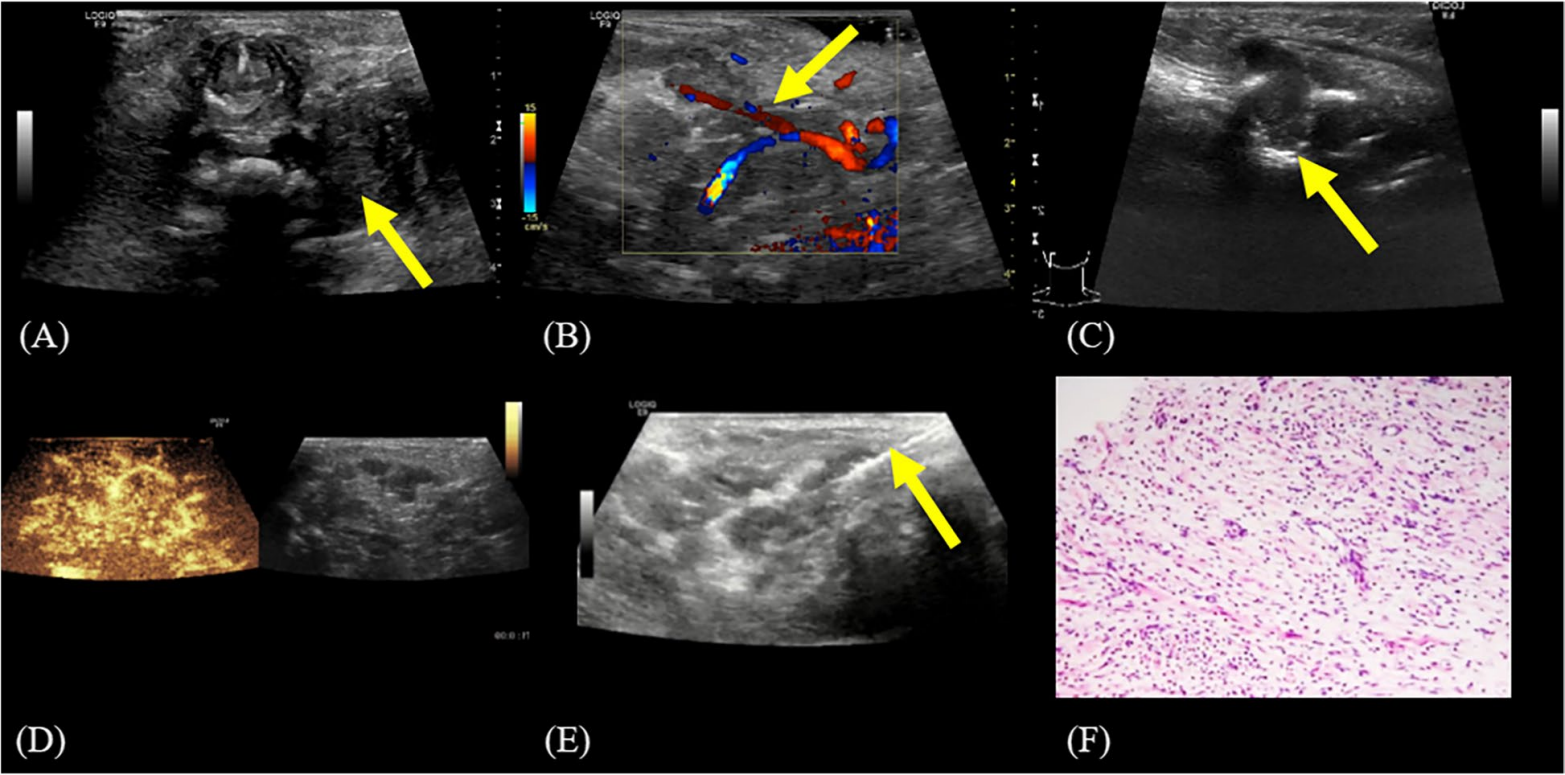
Ultrasound and pathological images. **A** Transverse image of the irregular hypoechoic mass in the deep facial space beside the left cervical spine (arrow); **B** Image of the mass shows bead-like echo, encircling the external carotid artery and its branches; **C** Bone destruction is observed in the left mandible (arrow); **D** Contrast-enhanced ultrasound shows rapid hyperenhancement and slow clearance within the mass, without any obvious non-enhancement area; **E** Ultrasound-guided puncture of the mass (arrow); **F** Puncture tissues mainly consist of spindle cells

The ultrasound showed a large irregular and substantial hypoechoic mass in the deep facial space besides the left cervical spine, around 6.6 × 6.0 × 5.5 cm. The lower edge of the mass was equal to the level of the thyroid gland, the inner upper edge reached the level of the skull base, and the upper edge had unclear boundaries with the submandibular gland and parotid gland. Echo within the mass was uneven. The mass wrapped around the entire extracranial segment of the internal carotid artery to the cranial base level, external carotid artery and branches. The jugular veins were compressed to become narrow. Color Doppler ultrasound revealed dotted blood flow signals within the mass. Contrast-enhanced ultrasound displayed rapid hyperenhancement with slow contouring within the mass, without obvious non-enhancement areas (Fig. 3A-D). Ultrasound diagnosis showed large substantial space occupying lesions on the left cervical and maxillofacial region, and thus neurofibromatosis was considered.

Due to the compression of the trachea by the cervical mass, the child was urgently intubated and ventilated with a ventilator. After 2 days of observation, ultrasound-guided puncture of the mass was performed (Fig. 3E). The pathological findings of the swelling puncture biopsy (hematoxylin-eosin staining) showed that the puncture tissue consisted mainly of spindle cells. The nuclei were oval or short spindle-shaped, with inconspicuous heterogeneity, and no obvious nuclear division was seen. The cytoplasm of the cells was lightly stained or lightly pink stained (Fig. 3F). Immunohistochemical analysis of tumor cells showed S-100 protein (+) and CD34 (+). Histologic examinations were consistent with neurofibromatosis. In light of the child’s medical history, NF1 was suspected. Then whole exome genetic test was conducted on her parents and the child. The results showed a heterozygous variant in their NF1 gene with the mutation locus c.2540T > C (p.L847P), which is a pathogenic mutation. As no mutation at this locus was found in either of her parent, this was a spontaneous germline mutation. Given the extent of the lesion and the low tolerability of the neonate, the possibility of complete surgical resection was low and the risk was high, so the child received conservative treatment temporarily. The child was discharged for family reasons and treated outside hospital, where he survived 6 months, and the family refused to provide treatment details.

### Discussion and conclusions

NF1 refers to germline mutation of the NF1 gene originating from chromosome 17q11.2. The mutation can be de novo or familial, characterized by autosomal dominant inheritance. Approximately 50% of NF1 patients are spontaneously mutated [4]. This has no family history of NF1 and is a spontaneous mutation. CALMs are early clinical features of NF1 [5]. According to the uniform diagnostic criteria of National Institutes of Health (NIH) for NF1 in 1988, six or more CALMs are diagnostic [6]. Generally, normal people also carry 1–3 CALMs sometimes, which could explain the coffee milk spots on the father.

In the past 3 decades, there have been few reports on neonatal NF1 (Table 1) [7–14]. Subcutaneous neurofibroma and plexiform neurofibroma (PNF) are present in 30–50% of NF1 patients. Usually congenitally benign, PNF grows in a reticular pattern to replace normal tissue [15]. PNF can be limited, nodular, or diffuse and seen in the paravertebral region of the trunk (31%), head & neck (31%) and extremities (25%) [5]. Previous ultrasound reports showed that PNF was an irregularly lobulated hypoechoic mass, which ran along the nerve axis on the longitudinal image diagram and displayed target ring sign on the transverse image, with high echo in the center and low echo around. Cystic changes can be seen in about 70% of the masses. However, the Color Doppler flow signals can have many different presentations that have no specificity [16]. The ultrasonography findings in this case were consistent with previous reports. In addition, after the literature review, we found that this case was the first report of using contrast-enhanced ultrasound and ultrasound-guided puncture biopsy to diagnose neurofibromatosis in the left maxillofacial and cervical region. The ultrasound contrast enhancement pattern with quick-in-and-out has been previously reported in a 35-year-old male with non-NF1 pancreatic neurofibroma and a 4-year-old with a giant retroperitoneal neurofibroma [17]. The enhancement pattern in this child was quick in-slow out. The exact cause of such discrepancy could not be demonstrated due to the lack of big data statistics, but this case has accumulated experience for this rare disease.

**Table 1:** Reports on Neonatal NF1 in the past 30 years

Author	Article Year	Age at diagnosis (d)	Gender	Family history	Presence of CALMs	Location of neurofibroma	Treatment
Berthin C et al. [7]	2019	21d	Male	Unknown	Yes	Eye	Unknown
Sadhukhan M et al. [8]	2017	8d	Female	Unknown	Yes	Retropharyngeal and paraspinal	Taking MEK inhibitors
Day KM, et al. [9]	2020	1d	Female	Unknown	Unknown	Neck	Microlaryngoscopy, bronchoscopy and tracheotomy plus trametinib chemotherapy
Goyal S et al. [10]	2014	6d	Female	Yes	No	Eye	Local excision
Hatanaka K et al. [11]	2013	1d	Male	No	Yes	Posterior mediastinum	Surgery
Colás-Tomás T et al. [12]	2010	1d	Male	Yes	Yes	Posterior ocular	Ahmed glaucoma flap implantation
Stewart H et al. [13]	2008	1d	Female	No	No	Diffuse, most severely involving the head and neck, and the mass found involving almost every tissue at autopsy	Died 42 h after birth
Payne MS et al. [14]	2014	1d	Female	No	None at birth, present at 10w	Paracervical spine	Local excision

PNF usually grows rapidly in early childhood at a volume of ≥ 20% per year [4]. In this case, the mass was large enough to compress the trachea, and thus tracheal intubation was required. This case suggested that the diagnosis of rare diseases should be made with caution and suspicion. Early diagnosis and treatment may improve the long-term prognosis [9].

Neonatal NF1 is rare. Diagnosis is more challenging if it is non-familial inheritance. In this case, the left mass was large enough to compress the airway and surround the blood vessels. Life-threatening conditions such as respiratory and cardiac arrest could occur at any time. Ultrasonic examination, which is convenient and multi-directional, is particularly necessary for early diagnosis. With a high success rate, ultrasound-guided aspiration biopsy has provided a reliable basis for the diagnosis of neurofibromatosis.

Maxillofacial and cervical mass in neonates accompanied by CALMs should consider NF1. The combination use of contrast-enhanced ultrasound and ultrasound-guided puncture biopsy can ensure the diagnosis of this disease.

## Data Availability

The datasets used and analysed during the current study are available from the corresponding author upon reasonable request.

## Abbreviations

NF1: Neurofibromatosis Type 1
CALMs: Cafe-au-lait macules
CNS: Central nervous system
NIH: National Institutes of Health
PNF: Plexiform neurofibroma
